# Scrub Typhus Co-Infections in a Young Boy With Varicella and Malaria: A Rare Case Report

**DOI:** 10.7759/cureus.24723

**Published:** 2022-05-04

**Authors:** Khushbu Pandey, Aroop Mohanty, Monika Matlani, Mamta Ahir, Vivek Hada

**Affiliations:** 1 Clinical Microbiology, All India Institute of Medical Sciences, Gorakhpur, Gorakhpur, IND; 2 Clinical Microbiology, Vardhman Mahavir Medical College and Safdarjung Hospital, Delhi, IND

**Keywords:** doxycycline, coinfection, malaria, varicella, scrub typhus

## Abstract

Scrub typhus is one of the leading causes of acute febrile illness of unknown origin in India. Though several co-infections of other vector-borne diseases have been described in the literature, few such cases have been described in children. As such, it is challenging to diagnose scrub typhus alone and becomes that more complicated when a varicella infection precedes it. This is the first reported case where scrub with varicella infection also had concomitant malaria. In such cases, prompt diagnosis and initiation of the correct drug are imperative. Here we describe a six-year-old child with a past history of varicella infection and co-infected with scrub typhus and malaria.

## Introduction

Scrub typhus is a life-threatening zoonotic disease caused by Orientia tsutsugamushi, an obligate, intracellular, Gram-negative bacteria isolated in 1930 [1]. The disease derives its name from the scrub vegetation where its vector, Leptotrombidium mite, resides. It is endemic to a geographically distinct region named the tsutsugamushi triangle, extending from northern Japan and eastern Russia in the north, to Australia in the south and Pakistan and Afghanistan in the west. Several cases have been reported from India, initially from Southern India and later on from the Himalayan belt spreading from Jammu and Kashmir in the North to Arunachal Pradesh in the East [2,3]. Scrub typhus can present either as an uncomplicated mild febrile illness with or without a rash or as a complicated febrile illness with organ dysfunction and fatality. Of late, there has been an increase in the number of co-infections with scrub typhus. The most concerning part about such cases is the rapid deterioration seen due to various organ complications. Due to the wide variation in clinical manifestations, it is challenging to diagnose scrub typhus singly and leave it alone when associated with other infections. Here we report a young boy with varicella and malaria who had scrub typhus co-infection.

## Case presentation

A six-year-old male child presented to the pediatrics outpatient department (OPD) with fever for nine days, severe headache, bleeding from gums for five days, and loose stools with vomiting for the last two days. He also gave a history suggestive of pleomorphic vesicular-papular rash 10-12 days back and a similar family history in other members recently. The fever was associated with chills and rigors and persisted even after the healing of scars. On examination, he was moderately built with healed scabs all over the body. Physical examination revealed a febrile child with pallor, icterus, and tender lymphadenopathy of inguinal and axillary regions. The rest of the systemic examination was within normal limits. As the fever persisted even after the varicella lesions had healed well, he was further investigated for other causes of fever. Blood samples were collected and sent for routine investigations. The blood count was normal, but the liver enzymes were raised. Both alanine aminotransferase (ALT: 415 IU/L) and alkaline phosphatase (ALP: 420 IU/L) were markedly elevated along with raised bilirubin (3 mg/dl). The blood culture sent did not show any growth. A peripheral blood smear showed asexual forms of Plasmodium vivax, also positive with a rapid diagnostic test (RDT). The patient was immediately started on injectable artesunate 2.4 mg/kg IV but did not show any signs of improvement even after 48 hours.

At this point, other causes of febrile illnesses like dengue, leptospirosis, enteric fever, and scrub typhus were considered and investigated. Immunochromatography test (ICT) for scrub typhus (Bioline Tsutsugamushi Kit, Standard Diagnostics, Republic of Korea) was positive which was further confirmed by ELISA (Scrub Typhus Detect™ IgG and IgM ELISA System, InBios International, USA) (Table 1). Rest all tests came out to be negative. Injectable doxycycline (2.2 mg/kg IV every 12 hours) was started on an urgent basis along with injectable artesunate, which was continued as before. The patient responded to doxycycline and, within 24 hours, became afebrile. His condition improved gradually, and he was discharged after 48 hours.

**Table 1:** Laboratory parameters.

Parameter	Results	Reference Range
WBC Count	14 x 10^3^/mcL	4-11 × 10^3^/mcL
Alanine Aminotransferase (ALT)	415 IU/L	0-35 IU/L
Alkaline Phosphatase (ALP)	420 IU/L	30-120 IU/L
Total Bilirubin	3 mg/dl	0.3-1.2 mg/dL
Blood Culture	Sterile	NA
ELISA for Scrub Typhus	Positive	NA
Rapid Diagnostic Test (RDT) for Malaria	P. falciparum Positive	NA

## Discussion

The Rickettsial diseases, which were once thought to have disappeared from India, are now making a strong comeback and are reemerging in different parts of the country. Scrub typhus is now the most common Rickettsial disease in the Indian sub-continent and is usually presented singly. Co-infections of scrub typhus are very rare in children and complicate the course and management of the disease. The usual co-infections seen in children with scrub typhus include leptospirosis, dengue, and malaria [4,5]. Leptospirosis, along with scrub typhus, can be explained as both infections are spread by rodents. Malaria and dengue are very frequently seen in areas with heavy rainfall, where water stagnation favors mosquito breeding and the displacement of rodents from their natural habitat. Scrub typhus with a varicella infection is a rare co-infection, and only a few cases have been published in literature till now [6,7]. Fever in varicella infection generally lasts for three to five days. In our case, the boy was brought to the hospital as the fever persisted even after the healing of the scars, and also other family members recovered completely. The most common causes of fever in varicella are super-added infections of the skin like Streptococcus pyogenes and Staphylococcus aureus but were excluded in our case as the blood culture came out sterile. Routine blood investigations detected malaria, but despite starting the patient on anti-malarial drugs, the fever did not touch the baseline. As most of the body was covered with healed scabs, it is possible that an eschar present could have been missed during the physical examination, and thus scrub typhus was never considered initially. A breach in the skin in varicella infection might actually facilitate the entry of the mite without causing much inflammation and no eschar formation. In our case, the boy at presentation had recovered from varicella and was not treated for the same. Continuous fever along with headache and vomiting pointed towards a co-infection, and it was later found that he had malaria along with scrub typhus. He recovered completely after being administered doxycycline, which finally improved his condition substantially. Scrub typhus is endemic in this part of the country. It should be considered a differential diagnosis in any patient with unexplained fever (fever of unknown origin and acute febrile illness) [8]. Prompt diagnosis of co-infections will help us considerably decrease the associated mortalities due to the primary infection.

## Conclusions

Scrub typhus is a reemerging disease in our country and has been prevalent in many parts of the country, with maximum prevalence in the Himalayan states. In the initial cases reported in the literature, all the cases were presented as isolated, but as the years have passed, various other concomitant infections have emerged. This case illustrates the importance of having a differential diagnosis in mind when looking at a case of pyrexia of unknown origin, especially in endemic areas for scrub typhus. We should always be on the lookout for scrub typhus, even in cases of malaria, dengue, leptospirosis, and typhoid.
